# Morphometric analysis of inflammation in bronchial biopsies following exposure to inhaled diesel exhaust and allergen challenge in atopic subjects

**DOI:** 10.1186/s12989-016-0114-z

**Published:** 2016-01-13

**Authors:** Ali Hosseini, Jeremy A. Hirota, Tillie L. Hackett, Kelly M. McNagny, Susan J. Wilson, Chris Carlsten

**Affiliations:** 1 Department of Medicine, Division of Respiratory Medicine, Chan-Yeung Centre for Occupational and Environmental Respiratory Disease, University of British Columbia, Vancouver, BC V5Z 1M9 Canada; 2 Institute for Heart and Lung Health, University of British Columbia, Vancouver, BC V6Z 1Y6 Canada; 3 Biomedical Research Centre, University of British Columbia, Vancouver, BC V6T 1Z3 Canada; 4 Histochemistry Research Unit, Faculty of Medicine, University of Southampton, Southampton, S016 6YD UK; 5 The Lung Center, Vancouver General Hospital (VGH) – Gordon and Leslie Diamond Health Care Centre, 2775 Laurel Street, 7th floor, Vancouver, BC V5Z 1M9 Canada

**Keywords:** Particulate matter, Segmental allergen challenge, Airway inflammation, GMA immunohistochemistry, IL-4, ECP, Tryptase, CD4, Neutrophil elastase, CD138 (syndecan-1)

## Abstract

**Background:**

Allergen exposure and air pollution are two risk factors for asthma development and airway inflammation that have been examined extensively in isolation. The impact of combined allergen and diesel exhaust exposure has received considerably less attention. Diesel exhaust (DE) is a major contributor to ambient particulate matter (PM) air pollution, which can act as an adjuvant to immune responses and augment allergic inflammation. We aimed to clarify whether DE increases allergen-induced inflammation and cellular immune response in the airways of atopic human subjects.

**Methods:**

Twelve atopic subjects were exposed to DE 300 μg.m^−3^ or filtered air for 2 h in a blinded crossover study design with a four-week washout period between arms. One hour following either filtered air or DE exposure, subjects were exposed to allergen or saline (vehicle control) via segmental challenge. Forty-eight hours post-allergen or control exposure, bronchial biopsies were collected. The study design generated 4 different conditions: filtered air + saline (FAS), DE + saline (DES), filtered air + allergen (FAA) and DE + allergen (DEA). Biopsies sections were immunostained for tryptase, eosinophil cationic protein (ECP), neutrophil elastase (NE), CD138, CD4 and interleukin (IL)-4. The percent positivity of positive cells were quantified in the bronchial submucosa.

**Results:**

The percent positivity for tryptase expression and ECP expression remained unchanged in the bronchial submucosa in all conditions. CD4 % positive staining in DEA (0.311 ± 0.060) was elevated relative to FAS (0.087 ± 0.018; *p* = 0.035). IL-4 % positive staining in DEA (0.548 ± 0.143) was elevated relative to FAS (0.127 ± 0.062; *p* = 0.034). CD138 % positive staining in DEA (0.120 ± 0.031) was elevated relative to FAS (0.017 ± 0.006; *p* = 0.015), DES (0.044 ± 0.024; *p* = 0.040), and FAA (0.044 ± 0.008; *p* = 0.037). CD138 % positive staining in FAA (0.044 ± 0.008) was elevated relative to FAS (0.017 ± 0.006; *p* = 0.049). NE percent positive staining in DEA (0.224 ± 0.047) was elevated relative to FAS (0.045 ± 0.014; *p* = 0.031).

**Conclusions:**

In vivo allergen and DE co-exposure results in elevated CD4, IL-4, CD138 and NE in the respiratory submucosa of atopic subjects, while eosinophils and mast cells are not changed.

**Trial registration:**

URL: http://www.clinicaltrials.gov. Unique identifier: NCT01792232.

**Electronic supplementary material:**

The online version of this article (doi:10.1186/s12989-016-0114-z) contains supplementary material, which is available to authorized users.

## Background

According to the World Health Organization (WHO), 7 million deaths were attributed to air pollution in 2012, representing 1 in 8 deaths worldwide and making air pollution the biggest single environmental health risk [1, 2]. The mortality was more than twice the 2008 estimates, confirming that air pollution is increasingly taking a toll on human health [2]. The WHO results are substantiated by numerous epidemiological studies that have established a significant association between exposure to ambient air particulate matter (PM) and increases in mortality and morbidity due to cardiovascular and respiratory diseases [3–5].

Asthma is a major public health problem and it is estimated that 300 million people suffer from asthma around the globe, with 250,000 annual deaths and more than 2 million annual emergency room visits in the U.S. [6, 7]. Asthma is commonly defined as a chronic inflammatory condition characterized by airway inflammation, reversible airway obstruction, and increased airway responsiveness leading to symptoms such as wheezing, coughing, shortness of breath, and chest tightness [8]. Airway inflammation in asthmatic patients may stem from a hypersensitivity of the respiratory tract to triggers like allergens and air pollutants resulting in accumulation of chronic inflammatory cells in the airway wall [9]. Specific cures for asthma remain elusive and therefore, in order to develop new therapeutic strategies and inform health policy on air quality, we require a greater understanding of the mechanisms behind how environmental exposures can trigger asthma attacks [10].

Toxicological studies have shown that ambient airborne PM can induce the production of cytokines and oxidants that initiate airway inflammation [11]. PM may have direct effects on the pulmonary system, including induction of an inflammatory response, exacerbation of existing airway disease or impairment of pulmonary defense mechanisms [12]. Epidemiologic reports have indicated that there is a higher prevalence of asthmatic and allergic symptoms in people who live in close proximity to major roads relative to those in more distant locations [13–15]. Diesel exhaust (DE) is a main contributor to ambient PM air pollution [16]. It has been suggested that exposure to DE can trigger T-helper type 2 (Th2) immune responses which are directly associated with the development and exacerbation of allergic asthma [17]. Consistent with observational studies, animal and human nasal models have demonstrated that DE can act to augment allergic immune responses [18–21].

The chronic airway inflammation in allergic asthma is characterized by activation of mast cells, type 2 innate lymphoid cells (ILC2), T cells and infiltration of activated eosinophils and basophils [22]. Inhalation of allergen, results in Th2 cell activation and secretion of inflammatory cytokines such as interleukin (IL)-4, IL-5, IL-9 and IL-13, which are considered to play an important role in the mucus hyper-secretion, thickening and contraction of airway smooth muscle in atopic asthmatic patients [22–25]. Asthma is a phenotypically heterogeneous disorder and, over the years, many different clinical subtypes of asthma have been described [26] and classified by trigger or symptoms such as allergic, non-allergic, exercise-induced and cold-induced [27]. In our current study, we have focused on classic allergic asthma, and the study design and interpretation followed accordingly. It has been hypothesized that environmental allergens impose a greater effect in the presence of DE exhaust but the exact mechanism behind this synergy is still not clear. Our study investigated the impact of DE exposure on allergen-induced airway inflammation as assessed in the submucosa of bronchial biopsies. Our hypothesis was that allergen exposure results in an increase in airway inflammation in the submucosa that was synergistically increased with DE exposure. We used a blinded crossover study design in atopic human subjects with segmental allergen challenge following controlled exposure to either filtered air or freshly generated DE. Our results demonstrate that controlled allergen exposure resulted in no increases in submucosa mast cells or eosinophils, 48 h post-exposure, in either filtered air or DE exposed conditions. In contrast, combination allergen and DE exposure resulted in an increase in submucosa CD4, IL-4, CD138 and NE relative to filtered air and saline. Our results suggest a role for CD4^+^ and Th2 immune responses in airway inflammation in response to combination exposures that may not be observed in single exposure studies in atopic subjects.

## Results

### Subject characteristics

Study subject gender, age, height, weight, body mass index (BMI), forced expiratory volume in 1 s (FEV_1_), methacholine PC_20_, and allergen used for segmental allergen challenge are described in Table 1. The study involved samples from 7 female subjects and 5 male subjects. Samples from all 12 subjects were used for IL-4 immunostaining and morphometric analysis. For tryptase, eosinophil cationic protein (ECP), CD4, CD138 and neutrophil elastase (NE) staining, two bronchial biopsies did not contain sufficient submucosa area, resulting in only 10 subjects available for comparison across the four experimental conditions for these endpoints.

**Table 1:** Subject characteristics

Subject	Gender	Age (years)	Height (cm)	Weight (kg)	BMI	FEV_1_ (% of pred)	Methacholine PC_20_ (mg/mL)	Positive SPT
1	F	20	158	54.6	22	104	13.85	HDM
2	F	31	173	70	24	113	>16	HDM
3^a^	M	32	161	68	26	123	30.74	Pacific Grasses
4	F	34	157	55	22	79	0.23	Pacific Grasses
5	M	27	178	70	22	105	>128	HDM
6	F	25	173	73	25	117	>128	HDM
7^a^	M	27	186	85	25	107	87.64	Pacific Grasses
8	F	46	165	65	24	63	>128	HDM
9^a^	F	31	146	50	23	103	0.26	Birch
10	M	28	176	90	29	100	19.12	Pacific Grasses
11^a^	M	23	169	83	29	104	2.41	Pacific Grasses
12	F	23	172	96	32	101	54.32	HDM
Mean (SD)		29 (7)	168 (11)	72 (15)	25 (3)	102 (16)		

*M* male, *F* female, *BMI* body mass index, *FEV*_*1*_ forced expiratory volume in one second, *% of pred* percentage of predicted, *PC*_*20*_ provocative concentration causing a 20 % fall in FEV_1_, *SPT* skin prick test, *HDM* house dust mite allergen

^a^Previous smoker

### Submucosal changes within bronchial biopsies induced by single exposure or co-exposure

#### Tryptase and ECP are unchanged by the combination of diesel exhaust and allergen (DEA)

**Fig. 1:**
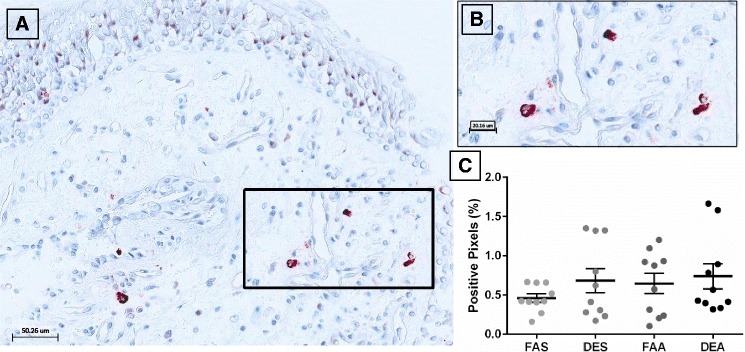
Immunohistochemical staining of tryptase-positive mast cells in human bronchial submucosa tissue. **a** Representative 20X image of positive staining using mAb AA1 for tryptase with positive staining in red with Mayer’s hematoxylin counterstain in blue from a subject exposed to FAA. **b** Zoom region (40X) highlighted in panel **a** black box. **c** Positive pixel count quantification of submucosa region for tryptase staining. Data are expressed as mean ± SEM. *n* = 10 for each experimental condition

Analysis of tryptase-positive staining in bronchial mucosa revealed distinct and strong granular cytoplasmic staining in cells with isolated observations of extracellular staining (Fig. 1b). Quantification of the tryptase positively stained pixels, demonstrated no differences between any experimental conditions (Fig. 1c) although there was a trend to increased staining in the DEA (diesel exhaust + allergen) samples.

**Fig. 2:**
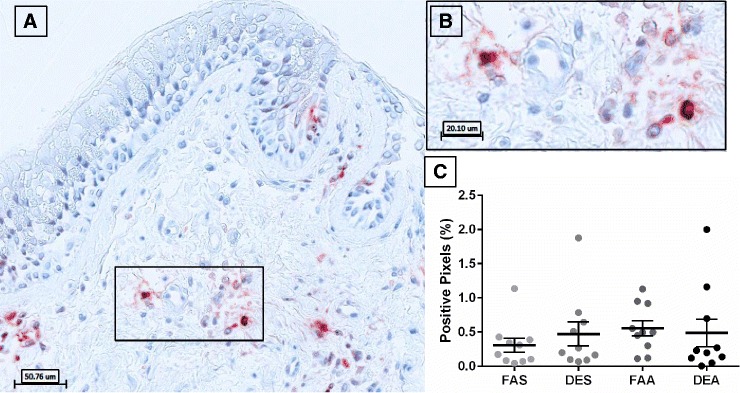
Immunohistochemical staining of ECP-positive eosinophils in human bronchial submucosa tissue. **a** Representative 20X image of positive staining using mAb EG2 for ECP with positive staining in red with Mayer’s hematoxylin counterstain in blue from a subject exposed to FAA. **b** Zoom region (40X) highlighted in panel **a** black box. **c** Positive pixel count quantification of submucosa region for ECP staining. Data are expressed as mean ± SEM. *n* =10 for each experimental condition

Analysis of ECP-positive staining in bronchial mucosa revealed distinct cytoplasmic localization in cells with pervasive observations of extracellular staining suggesting eosinophil degranulation (Fig. 2b). Quantification of the ECP-positive pixels, demonstrated no differences between experimental conditions (Fig. 2c).

### Neutrophil elastase is elevated by DEA

**Fig. 3:**
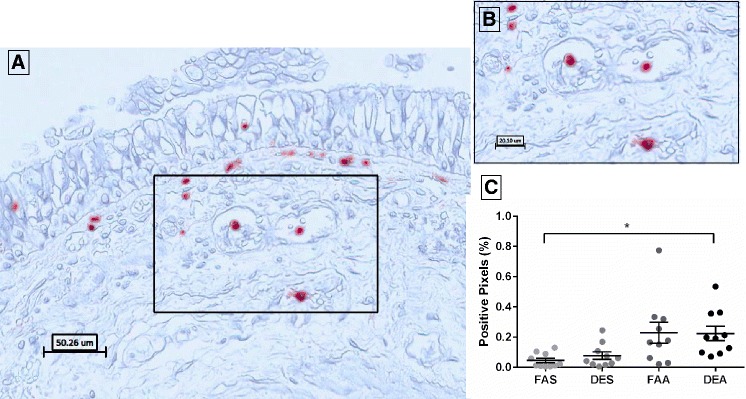
Immunohistochemical staining of elastase-positive neutrophils in human bronchial submucosa tissue. **a** Representative 20X image of positive staining using mAb NP57 for NE with positive staining in red with Mayer’s hematoxylin counterstain in blue from a subject exposed to FAA. **b** Zoom region (40X) as highlighted in black box of panel **a**. **c** Positive pixel count quantification of submucosa region for NE staining. Data are expressed as mean ± SEM. *n* = 10 for each experimental condition. *p < 0.05

Analysis of NE-positive staining in bronchial mucosa revealed distinct immunohistochemical localization of elastase in neutrophils with no observations of extracellular staining (Fig. 3b). Quantification of the NE-stained pixels demonstrated a significant increase (*p* = 0.031) in staining after DEA (0.224 ± 0.047) relative to FAS (0.045 ± 0.014). There were no significant differences between FAS vs. DES (0.077 ± 0.024, *p* > 0.999) or FAA (0.229 ± 0.069, *p* = 0.175) (Fig. 3c).

### CD138 positive cells are elevated by FAA and DEA

**Fig. 4:**
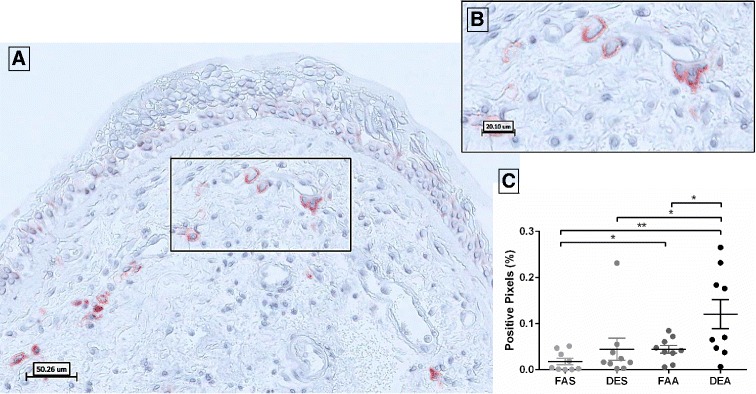
Immunohistochemical staining of CD138-positive plasma cells in human bronchial submucosa tissue. **a** Representative 20X image of positive staining using mAb B-A38 for CD138 with positive staining in red with Mayer’s hematoxylin counterstain in blue from a subject exposed to FAA. **b** Zoom region (40X) highlighted in black box of panel **a**. **c** Positive pixel count quantification of submucosa region for CD138 staining. Data are expressed as mean ± SEM. *n* = 9 for each experimental condition. *p < 0.05; **p < 0.01

Analysis of CD138-positive staining in bronchial mucosa revealed distinct membrane staining in plasma cells (Fig. 4b). Quantification of the CD138-positive pixels demonstrated a significant increase in staining after DEA (0.120 ± 0.031) relative to FAS (0.017 ± 0.006, *p* = 0.015), DES (0.044 ± 0.024, *p* = 0.040), and FAA (0.044 ± 0.008, *p* = 0.037). CD138 positive staining in FAA was elevated relative to FAS (0.017 ± 0.006; *p* = 0.049) (Fig. 4c).

### CD4 positive cells are significantly elevated by DEA

**Fig. 5:**
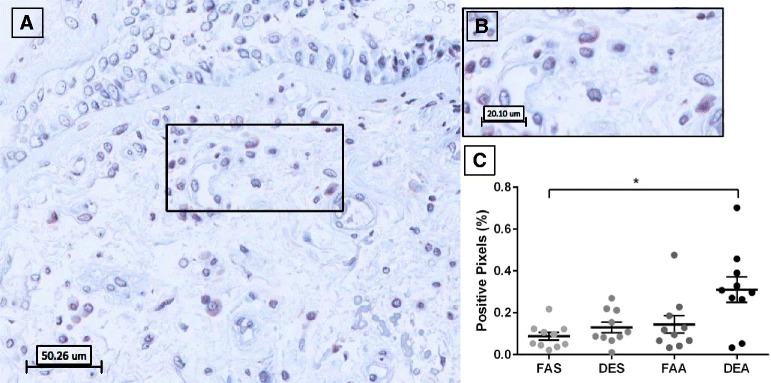
Immunohistochemical staining of CD4-positive T cells in human bronchial submucosa tissue. **a** Representative 20X image of positive staining using mAb 4B12 for CD4 with positive staining in red with Mayer’s hematoxylin counterstain in blue from a subject exposed to FAA. **b** Zoom region (40X) highlighted in black box of panel **a**. **c** Positive pixel count quantification of submucosa region for CD4 staining. Data are expressed as mean ± SEM. *n* = 10 for each experimental condition. *p < 0.05

Analysis of CD4-positive staining in bronchial mucosa revealed distinct membrane staining in T cells (Fig. 5b). Quantification of the CD4-positive pixels demonstrated a significant (*p* = 0.035) increase in staining after DEA (0.311 ± 0.060) relative to FAS (filtered air + saline [control (vehicle) for allergen]; 0.087 ± 0.018).

### IL-4 expression is elevated by DEA

**Fig. 6:**
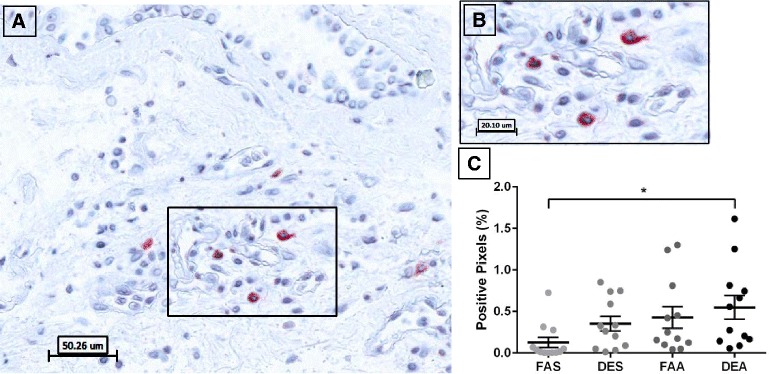
Immunohistochemical staining of IL-4-positive cells in human bronchial submucosa tissue. **a** Representative 20X image of positive staining using mAb 4D9 for IL-4 with positive staining in red with Mayer’s hematoxylin counterstain in blue from a subject exposed to FAA. **b** Zoom region (40X) highlighted in black box of panel **a**. **c** Positive pixel count quantification of submucosa region for IL-4 staining. Data are expressed as mean ± SEM. *n* = 12 for each experimental condition. *p < 0.05

Analysis of IL-4-positive staining in bronchial mucosa revealed distinct immunohistochemical localization of IL-4 in cells with no observations of extracellular staining (Fig. 6b). Quantification of the IL-4-stained pixels, demonstrated a significant increase (*p* = 0.034) in staining for DEA samples (0.548 ± 0.143) relative to FAS samples (0.127 ± 0.062). There were no significant differences between FAS vs. DES (0.353 ± 0.088, *p* = 0.086) or FAA (0.426 ± 0.130, *p* = 0.150).

Finally, Pearson correlation coefficients matrix was performed for each of the endpoints versus each other (Additional file 1: Tables S1 and S2). No endpoints were significantly correlated under DEA, suggesting potential distinctive biology associated with the endpoints induced by DEA. NE and CD138 were positively correlated (r = 0.76, *p* < 0.01) after DES. After FAA, tryptase and CD138 were positively correlated (r = 0.64, *p* < 0.04) and ECP and IL-4 were also positively correlated (r = 0.61, *p* < 0.05).

## Discussion

To our knowledge this is the first blinded crossover human study using controlled exposures to a combination of DE and allergens to investigate the lower airway inflammatory responses in bronchial biopsies in atopic individuals. Considerable evidence suggests that that the effects of DE and allergens are synergistic [28]. We provide further evidence for synergy by demonstrating that a combination of DE and allergen (but not DE or allergen alone) results in augmented CD4, IL-4, NE and CD138. Our results suggest that studies examining allergen or air pollution in isolation, including those related to drug development, may underestimate the real-world impact of heterogeneous environmental exposures on airway inflammation in atopic humans [29–31]. Our results have implications for health policy aimed at protecting air quality for vulnerable populations [32], recognizing that the concentration of DE 300 μg.m^−3^ used in our study is most relevant in the context of occupational exposure in Western countries or the daily average level of PM in some developing countries in the world such as China and India [33, 34].

Mast cells are known to participate in allergic inflammation [35]. Previous studies have shown increases the number of mast cells in healthy volunteers at 6 h [36] and 18 h [37] after exposure to DE. Tryptase is the most plentiful granule constituent in mature and activated mast cells [38], thus we stained for tryptase as it is a reliable biomarker of mast cell presence and activation. Basophils are the only other cell type that express tryptase, but their frequency is known to be considerably lower than that of in mast cells [39] and tryptase levels in human basophils are less than 1 % of those in tissue mast cells [40]. Our results demonstrate that 48 h following co-exposure to DE and allergen, there was no elevations in tryptase positive cells in the submucosa.

Eosinophils are another pro-inflammatory white blood cell type that differentiates from myeloid progenitors in the bone marrow; mature eosinophils travel through blood vessels to mucosal surfaces throughout the body [41]. We examined ECP as a selective marker for eosinophils, as this protein is largely restricted to these cells; there is evidence that ECP can be detected in neutrophils [42, 43], but rarely and weakly [44]. Staining ECP (rather than MBP or EPO) as a markers of intensity of eosinophilic inflammation of the airways in allergic diseases [45], allowed us to compare our results with other DE controlled exposure studies [46, 47].

Our data demonstrate that in atopic individuals, combination exposure (DE followed by allergen) does not impact ECP expression in bronchial submucosa. This is consistent with a previous controlled DE exposure study that showed no significant increase in the count of tissue eosinophils at 18 h post-exposure [37], although studies examining bronchoalveolar lavage [48] and induced sputum [47] have shown increases at 6 h post-exposure.

We chose a later time point 48 h for sampling tissue biopsies, thus there is potential that early inflammatory cell recruitment and activation was missed due to resolution of the inflammatory event. A previous study utilizing segmental allergen challenge was aimed to elucidate the time course of inflammatory events in allergic airway diseases. They have shown that the level of tryptase in BAL increases immediately within 12 min following the segmental allergen broncho-provocation with ragweed in allergic rhinitis patients, but this signal was resolved by 48 h. In contrast, ECP levels were only increased at the late time point [49]. This leads us to speculate that in our tissue biopsies, ECP was released from the tissue eosinophils and by 48 h was measureable only in BAL [50].

Neutrophilic inflammation is associated with progression and the development of chronic respiratory diseases, such as severe asthma [51]. Neutrophils are granulocytes and one of the first responders to the environmental insults and migrate to the site. In neutrophils, neutrophil elastase (NE) contributes mainly to digestion of ingested foreign particles, chemotaxis, infiltration and tissue remodeling by degrading connective tissue proteins such as elastin and collagens [52]. Exposure to DEP is associated with accumulation of neutrophils and it has been shown that DEP can activate neutrophils and augment the expression of NE and other mediators of tissue destruction [53, 54].

We demonstrate that NE expression is increased by DEA, suggesting neutrophilic inflammation is induced by the combined exposure to DE and allergen. Our data are consistent with our own study of combined exposure [50], in which BAL neutrophils were increased by DE plus allergen, but contrast somewhat with previous human controlled studies that reported increased neutrophils associated with DE in various compartments at multiple timepoints through 24 h post-exposure [55], since we showed so similar effect (assessing DES alone) at 48 h.

CD138 (syndecan-1) is a cell surface proteoglycan and predominantly is expressed on mature plasma cells and weakly in epithelial cells. CD138 is highly sensitive and specific marker for identification of plasma cells and plasma cell differentiation. CD138 modulates cell growth, differentiation, adhesion and migration, thus plays important roles in the regulation of inflammatory responses [56].

Aggregation of CD138^+^ plasma cells were identified in pulmonary fibrosis [57] and in the lung submucosa of severe asthmatics with increased inflammatory lymphocytes infiltrates [58]. Increased frequencies of CD138^+^ IgE^+^ cells were detected in the lamina propria of the nasal mucosal biopsies from allergic patients [59]. Also the number of CD138^+^ IgE^+^ cells was positively correlated with the IgE serum titres in atopic individuals [60]. Exposure to environmental allergens triggers Th2 cells differentiation and production of IL-4 and IL-13 which proliferate and differentiate B cells into plasma cells and switch to IgE synthesis [61]. It has been shown that inhalation allergen challenge significantly increases the number of CD138^+^ IgE-secreting cells in murine lungs [62]. CD138 immunostaining is proven to be an excellent indicative of IgE^+^ cells in the lung tissue [63]. CD138^+^ cells were detectable in bronchial biopsies at 24 h but not in BAL after segmental allergen challenge in atopic asthmatics [64].

We demonstrate that CD138 expression is increased in DEA vs. FAS, DES and FAA, suggesting plasmacytosis is induced by DE added to allergen. CD138 expression in FAA was elevated relative to FAS, suggesting the effect of allergen challenge in submucosal plasmacytosis. Our data are consistent with a previous human nasal model that have confirmed that co-administration of DE particles and allergen stimulate an increase in level of allergen specific IgE in nasal lavage samples [21] and a trend in our own study of BAL from DE-allergen co-exposure [50].

CD4^+^ T-cells play an essential role in adaptive allergic immune responses. CD4 is a transmembrane glycoprotein selectively expressed on the surface of helper T-cells and plays an important role in the regulation of T-cell signalling and its functional consequences [65]. Following activation, naive CD4^+^ T-cells differentiate into one of the sub-types of T-helper cells: Th1 [66], Th2 [67], Th9 [68], Th17 [69], or Th22 [70], depending on the nature of antigen and the cytokines present in the surrounding milieu [71]. Th2 cells secrete IL-4, IL-5, IL-6, IL-9, IL-10 and IL-13 and induce eosinophil activation and differentiation; Th2 cells are more proficient B-cell helpers and can stimulate IgG1 and IgE production. Thus, they are well positioned to play substantial role in the pathogenesis of allergic inflammation. In the present work, we explored whether co-exposure to DE and allergen induced recruitment of CD4^+^ cells in human lung tissue. We demonstrate that the number of CD4^+^ cells significantly increased only in DEA vs. FAS (*p* = 0.035), which suggest an interaction between DE and allergen. In a single exposure model in healthy subjects, it has been previously argued that DE does not alter the number of CD3^+^, CD4^+^ and CD8^+^ lymphocytes in the bronchial tissue at 18 h post-exposure [72].

IL-4 is known to be an important cytokine in the development of allergic inflammation; it provides the first signal that initiates B-cell class switching to IgE production [73]. IL-4 can further enhance IgE-mediated immune responses by up-regulating the expression of low-affinity IgE receptor (FcεRII/CD23) on B-lymphocytes and macrophages and the high-affinity IgE receptor (FcεRI) on mast cells [74, 75]. IL-4 induces the differentiation of naive T lymphocytes into Th2 cells which secrete more IL-4, IL-5, and IL-13, maintaining a suitable environment for further Th2 cells differentiation [76, 77]. IL-4 can also stimulate the expression of vascular cell adhesion molecule-1 (VCAM-1) on endothelial cells, which leads to enhanced migration of T-cells, eosinophils, macrophages and mast cells to inflamed tissue [78]. To for Th2 cells but IL-4 is a signature cytokine of type 2 immunity. IL-4 is known as a positive feedback cytokine for Th2 cell differentiation that stimulates the differentiation of naive CD4^+^ cells into IL-4-secreting Th2 cells [79, 80]. It has been shown that human mast cells are one of the major sources of IL-4 in the skin, nasal and bronchial tissue [81–83]. IL-4 is able to induce the development of Th2 cells; thus, this stored and preformed IL-4 within mast cell granules has an important influence during the initiation and maintenance of the allergic immunological response. Basophils [84], naive T cells [85] and innate lymphoid cells [86] are also immediate source of IL-4 upstream of Th2 cells differentiation. IL-4 contributes to airway obstruction in asthma via the induction of mucus hypersecretion in mice and human cell lines, and increases the release of several pro-inflammatory cytokines such as IL-6, GM-CSF and eotaxin from human lung fibroblasts [87]. IL-4 is a major factor in the recruitment and activation of inflammatory cells that may contribute to inflammation and lung remodeling in chronic asthma [88].

We demonstrate that IL-4 expression is increased in DEA vs. FAS (*p* = 0.034), suggesting a Th2 immune response is induced by DE and allergen that is not observed with isolated (single) exposures. Our data are consistent with in vivo animal, in vitro*,* and human studies that have confirmed DE has strong pro-Th2 effect [89, 90]. Human nasal challenge studies have shown that co-administration of DE particles and allergen stimulate a Th2 immune response in nasal wash samples 4 days post-challenge [21, 91, 92]. There is one conflicting study that has shown no differences in expression of IL-4 in the bronchial submucosa but its authors mention that the bronchial tissue in their study was assessed at a single time point 6 h post-DE exposure [93] while most of the cytokine changes observed in previous human nasal studies and animal exposure studies were found 24 to 48 h post-exposure [94]. A previous study also evaluated the effects of diesel exhaust inhalation in enhancing allergic immunologic responses in lower airways [95]. Consistent with our results, they similarly found an increase in the IL-4 level (by 1.7-fold, which was close to statistical significance); they also found a non-significant elevation in the number of eosinophils in induced sputum due to DE exposure. However, there are some fundamental differences between their model and our current study a) their allergen challenge was performed by inhalation but we challenged our subjects segmentally, with saline control simultaneously, which confers some advantages and limitations; b) the diesel exhaust concentration that we used was ~300 μg.m^−3^ PM_2.5_ while theirs was ~100 μg.m^−3^; c) they analyzed sputum and blood that were acquired 22 h post-exposure while we analyzed endobronchial biopsies that were obtained 48 h post-exposure.

A primary strength of our study is that it is a double-blinded cross-over study that fundamentally eliminates typical confounding covariates, since each subject serves as his/her own control. Our study does have limitations, however. One limitation is generalizability. For example, given the specific gap (one hour) between inhalation exposure and segmental allergen, it is difficult to know whether similar findings would occur with simultaneous exposure or some other gap, but we effectively consider this “co-exposure” given that the particles from DE will remain in the airways for hours after inhalation and thus then be present when the allergen is inhaled (though, admittedly, the exact dynamics therein are unknown and an important future direction for our work). Another concern is whether airway changes relevant to our hypotheses can be induced by the bronchoscopy procedure itself. Investigative bronchoscopy and bronchial provocation challenge are commonly used techniques in airway inflammation research [96]. While FEV1 and PEFR dropped due to bronchoscopy with lavage and biopsies, both returned to baseline within 2 to 24 h [97, 98]. Accordingly, we doubt that significant inflammation from these procedures persists through 48 h.

## Conclusions

In summary, we have demonstrated for the first time that acute exposure to DE followed by segmental allergen challenge increases the submucosal recruitment of CD4 cells, CD138-positive plasma cells and expression of neutrophil elastase and IL-4 in the submucosa of atopic human subjects. Our study design and results suggest that experimental data from complex exposures can capture real-world exposures that enlighten our understanding of biology and maybe inform those concerned with public health and policy based on complex exposures.

## Methods

### Subject recruitment

Twelve atopic subjects (19–49 years old) were screened, informed of the protocol, procedures, and potential risk, and agreed to participate in the study (ClinicalTrials.gov identifier: NCT01792232). The UBC Research Ethics Board approved the study protocol and informed consent form. Allergic sensitization to house dust mite (HDM), birch and Pacific grasses were diagnosed by skin prick test. Birch-sensitive subjects were not studied in the birch season (February-April) and grass-sensitive subjects were not studied in the Pacific grasses season (May-August). Subject characteristics are described in Table 1.

### Exposure protocol

**Fig. 7:**
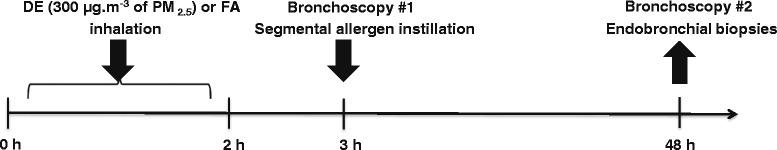
Schematic of exposure protocol. Study subjects were exposed to DE (300 μg.m^−3^ of PM_2.5_) or FA (filtered air) for 2 h. One hour post-exposure, a segmental allergen challenge was performed with allergen or saline vehicle in the right upper and middle lobe or left lingular lobe. Forty-eight hours post-exposure endobronchial biopsies were obtained via bronchoscopy. The process was repeated following a washout period of 1 month with exposure conditions reversed compared to the first visit

Each subject was exposed for 120 min to filtered air (FA; the control for diesel exhaust) or freshly generated diesel exhaust (DE PM_2.5_ 300 μg.m^−3^; Table 2) in a double-blinded crossover experiment, with the two distinct visits randomized and counter-balanced to order, with a four-week washout period between each condition (Fig. 7). In this study design, each subject serves as his/her own control.

**Table 2:** Inhaled exposure characteristics

Condition	PM_2.5_ (μg/m^3^)	Particle number (#/cm^3^)	CO (ppm)	NO (ppb)	NO_x_ (ppb)	NO_2_ (ppb)	NO_2_/PM# (μg/#)
FA	8.2 (6.9)	1750.4 (235.1)	2.8 (0.1)	25.3 (5.0)	71.1 (9.8)	45.9 (7.7)	4.9 × 10^−9^
DE	302.0 (30.5)	5.4 × 105 (6.4 × 104)	14.1 (2.0)	8665.5 (1287.1)	9185.3 (1366.1)	519.7 (118.6)	1.8 × 10^−9^

Values are presented as mean (SD)

*FA* filtered air, *DE* diesel exhaust

During the two-hour exposure, the subject was asked to exercise on a bicycle ergometer for a total of 30 min (2 × 15 min at ~60 rpm cadence and ~25 W of resistance) to increase ventilation-heart rate and mimic modest intermittent activity.

### Bronchoscopy procedure

#### Segmental allergen challenge (Bronchoscopy #1)

One hour following each exposure to DE or FA, segmental allergen challenge was performed through standard fiberoptic bronchoscopy procedure. Bronchoscopy was used to deliver a diluent-controlled solution of the positive skin prick allergen extract, at a concentration 10-fold lower than the dose producing a positive wheal ≥3 mm, into a right lower lobe segment. A 5 mL diluent control was delivered into a left lower lobe segment.

#### Endobronchial biopsies (Bronchoscopy #2)

The second bronchoscopy was done 48 h post-allergen challenge, endobronchial biopsy specimens (size ≤2 mm) were obtained from the same segments exposed to allergen or saline.

Following an approximately 1 month washout period, subjects returned and received the second two-hour exposure, followed by a bronchoscopy during which allergen and saline were administered to opposite lungs and different segments than those during the first exposure. Thus, endobronchial biopsies for each of the 4 different crossover conditions was created: 1) FAS: filtered air + saline, 2) DES: DE + saline, 3) FAA: filtered air + allergen and 4) DEA: DE + allergen.

### Bronchial biopsies processing

The endobronchial biopsies were immediately added to ice-cold acetone containing the protease inhibitors iodoacetamide (20 mM) and phenylmethylsulfonylfluoride (PMSF; 2 mM, Sigma, Oakville, ON) and fixed at −20 °C overnight (16–24 h). The next day, biopsies were transferred to fresh acetone and then to methyl benzoate (Sigma, Oakville, ON) for 15 min each at room temperature, before infiltration with glycol methacrylate (GMA) resin as previously described at Britten et al. [99].

### Glycol methacrylate acrylic resin (GMA) embedding

Glycol methacrylate acrylic resin (GMA) is a hydrophilic plastic resin which provides a number of advantages over frozen and paraffin-embedding techniques [100]. The JB-4 Embedding Kit (Polysciences, Warrington, PA) was used for embedding in GMA with some modification to its original manufacturer’s instructions described in details at Wilson et al. [100]. For polymerization of GMA resin, benzoyl peroxide was added to the airtight embedding capsule containing tissue sample and kept for 48 h at 4 °C to increase polymerization of GMA. The polymerized resin block was stored desiccated at −20 °C freezer until used for IHC staining.

### Biopsy quality evaluation

GMA blocks containing each biopsy were removed from embedding capsules and excess resin trimmed to form a trapezium shape around the tissue. Sections were cut at 2 μm using an ultra-microtome (Leica EM UC6 at JHRC Histology lab) and floated on distilled water (dH_2_O) and picked up onto 10 % poly-l-lysine (PLL)-coated slides (Fisher Scientific, Ottawa, ON). Slides were left on a hot plate to completely dry out, followed by addition of one drop of toluidine blue stain for 2 min following by drying and mounting in DPX (Sigma, Oakville, ON) for subsequent examination under light microscope to check biopsy quality. In order to qualify for immunohistochemical analysis the tissue section must have a minimum of 0.46 mm^2^ of submucosal tissue (lamina propria), excluding smooth muscle and glands [101]. If the section was found to be of poor standard, further sections were cut and reassessed. If no such level with acceptable histological standard was found in the biopsy, then the biopsy was excluded from further IHC analysis.

### Controls for Immunohistochemistry

Tonsil tissue samples removed from patients (kindly provided by Dr. Andrew Thamboo, Dept. of Otolaryngology (ENT) at St. Paul's Hospital) were used as positive control and staining with isotype-matched controls was used as negative control (Additional file 2: Figure S1). Endogenous peroxidase was blocked with a 100 μl of 30 % H_2_O_2_ solution in 10 mL of sodium azide and endogenous avidin and biotin were blocked using a commercially available kit from Vector Labs (Vector Laboratories, Burlington, Ontario). Colour was developed using a VECTASTAIN Elite ABC Kit (Vector Laboratories, Burlington, Ontario).

### Immunohistochemistry (IHC) on endobronchial biopsies

IHC was used to determine the number of CD4^+^, IL-4^+^ cells, CD138^+^ plasma cells, elastase-positive (NE^+^) neutrophils and activated (EG2^+^) eosinophils and tryptase-positive (AA1^+^) mast cells in the lamina propria in endobronchial biopsies (Table 3).

**Table 3:** Percent positivity for inflammatory biomarkers’ expression in the lung submucosa. Data are expressed as mean ± standard error of the mean (SEM)

Exposure condition	AA1	ECP	NE	CD138	CD4	IL-4
FAS	0.460 ± 0.053	0.308 ± 0.102	0.045 ± 0.014	0.017 ± 0.006	0.045 ± 0.014	0.127 ± 0.062
DES	0.681 ± 0.153	0.471 ± 0.175	0.077 ± 0.024	0.044 ± 0.024	0.045 ± 0.014	0.353 ± 0.088
FAA	0.646 ± 0.128	0.553 ± 0.109	0.229 ± 0.069	0.044 ± 0.008*	0.045 ± 0.014	0.426 ± 0.130
DEA	0.738 ± 0.159	0.487 ± 0.201	0.224 ± 0.047*	0.120 ± 0.031*^,^ **^,^ ***	0.045 ± 0.014*	0.548 ± 0.143*

*FAS* filtered air + saline, *DES* DE + saline, *FAA* filtered air + allergen, *DEA* DE + allergen, *AA1* tryptase, *ECP* eosinophil cationic protein, *NE* neutrophil elastase

*p < 0.05, compared with FAS; **p < 0.05, compared with DES; ***p < 0.05, compared with DEA

Semi-thin sections were cut at 2 μm from endobronchial biopsies and immunostained with monoclonal primary antibodies (Table 4). The immunostaining procedure followed has been described previously [100]. Briefly, endogenous peroxidase was blocked using a 100 μl of 30 % H_2_O_2_ solution in 10 mL of sodium azide and endogenous biotin were blocked using a commercially available kit from Vector Labs (Vector Laboratories, Burlington, Ontario). Mouse anti-human monoclonal antibodies were applied at appropriate dilutions (Table 4) and incubated with coverslip 20–22 h at room temperature. After washing with Tris-buffered saline (TBS, 3 × 5 min), the sections were incubated with the biotinylated rabbit anti-mouse secondary antibody (Dako, Burlington, Ontario) for 2 h. ABC (avidin-biotin complex) kit (Vector Laboratories, Burlington, Ontario) was applied after further washing with TBS (3 × 5 min), and incubated for 2 h. The positive staining were then visualized using AEC (3-amino-9-ethylcarbazole) chromogen kit (BioGenex, Fremont, CA) and counterstained with Mayer's Hematoxylin (Dako, Burlington, Ontario).

**Table 4:** List of primary monoclonal antibodies are used for IHC staining

Antibody	Marker	Cell	Concentration (μg.mL^−1^)	Catalog no.	Source
AA1	Tryptase	Mast cells	0.1	ab2378	Abcam Inc., Toronto, ON
EG2 (614)	ECP	Eosinophils	0.07	514-121	Diagnostics, Uppsala, SW
IL-4 (4D9)	IL-4	Th2, mast cells	20.0	211-44-134AX	Amsbio, Cambridge, UK
CD4 (4B12)	CD4	Helper T cells	8.0	M731001-2	Dako, Burlington, ON
CD138 (B-A38)	Syndecan-1	Plasma cells	1.0	MCA2459GA	AbD Serotec, Raleigh, NC
NE (NP57)	Elastase 2	Neutrophils	0.1	M075201-2	Dako, Burlington, ON

### Quantification of Immunohistochemistry

**Fig. 8:**
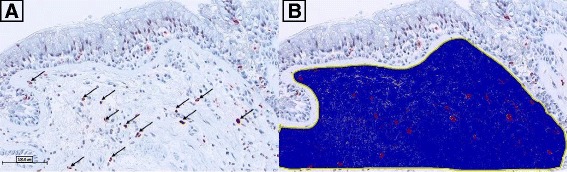
Demonstration of image analysis using Aperio® ImageScope™ software. A positive pixel count algorithm was used to quantify positive staining in the submucosa (*blue region*) of bronchial biopsies for tryptase, ECP, NE, CD138, CD4, and IL-4. The airway epithelium was not examined and positive staining in the epithelium was excluded from analysis. **a** Representative image of tryptase positive staining from a subject exposed to FAA, *Black arrows* denote positive staining in submucosa area that are selected by the positive pixel count. **b** Image from **a** with submucosa region selected by manual trace followed by positive pixel count recognition of tryptase stain (*red colour*) within submucosa region (*blue colour*)

IHC slides were scanned by Aperio ScanScope XT (Aperio Technologies, Vista, CA) at 40X magnification. Morphometric and immunohistochemical analysis were performed on the digital images (0.25 μm/pixel) using Aperio® ImageScope™ (version 11.2.0.780). The incorporated Positive Pixel Count (PPC) algorithm (version 9.1) was used to quantify inflammatory cells that stained positive (positive pixels) for each antibody of interest. To quantify positive pixels, a hue value of 0.0 (red) and hue width of 0.5 was used, and all the three intensity ranges (weak, positive, and strong) of staining were considered as positive (Fig. 8). The number of positive pixels was divided by the total number of pixels (positive and negative) in the analyzed area, and multiplied by 100, to calculate the percentage of positive pixels.

$$ \frac{\mathrm{Number}\;\mathrm{of}\;\mathrm{Positive}\;\mathrm{pixels}}{\mathrm{Total}\;\mathrm{number}\;\mathrm{of}\;\mathrm{Positive}\;\mathrm{pixels}+\mathrm{Negative}\;\mathrm{pixels}}\times 100\;\left(\%\;\mathrm{Positivity}\right) $$

For tryptase, ECP, NE, CD138, CD4 and IL-4, the amount of a positive stain in an image and the area of the submucosa were measured. The positive pixel counts were expressed as percent (%) positive pixel/mm^2^ in the submucosa.

### Statistical analysis

One-way repeated measures ANOVA with Bonferroni multiple comparison post hoc tests and Pearson correlation coefficients matrix were performed using GraphPad Prism® 6 software (GraphPad Software Inc., La Jolla, CA). Combined Robust regression and Outlier removal method (ROUT test) was used and an outlier in CD138 dataset was identified and removed. A p-value of <0.05 was considered statistically significant. Error bars shown represent the standard error of mean (±SEM).

## Additional files

Additional file 1: Tables S1 and S2. Pearson correlation coefficients matrix for inflammatory biomarkers’ expression in the lung submucosa after single or co-exposure to diesel exhaust and allergen. (PDF 213 kb)

Additional file 2: Figure S1. Immunohistochemical staining of positive and negative controls. A) Representative 40X image of positive staining using mAb AA1 for tryptase in human tonsil tissue; B) Representative 40X image of positive staining using mAb EG2 for ECP in human tonsil tissue; C) Representative 40X image of positive staining using mAb NP57 for neutrophil elastase in human tonsil tissue; D) Representative 40X image of positive staining using mAb B-A38 for CD138 in human lung tissue; E) Representative 40X image of positive staining using mAb 4B12 for CD4 in human tonsil tissue; F) Representative 40X image of positive staining using mAb 4D9 for IL-4 in human tonsil tissue; G) Representative 40X image of isotype control using mAb mouse IgG1 (0.07 μg.mL^−1^) in human lung tissue; H) Representative 40X image of isotype control staining using mAb mouse IgG1 (20.0 μg.mL^−1^) in human lung tissue. (PDF 482 kb)

## Abbreviations

BAL: bronchoalveolar lavage
BMI: body mass index
DE: diesel exhaust
DEA: diesel exhaust and allergen
DES: diesel exhaust and saline
ECP: eosinophil cationic protein
EPO: eosinophil peroxidase
FA: filtered air
FAA: filtered air and allergen
FAS: filtered air and saline
FEV_1_: forced expiratory volume in 1 s
GMA: glycol methacrylate resin
GM-CSF: granulocyte-macrophage colony-stimulating factor
HDM: house dust mite
IgE: immunoglobulin E
IHC: immunohistochemistry
MBP: major basic protein
NE: neutrophil elastase
PM: particulate matter
PM_2.5_: particulate matter less than 2.5 μm in diameter
Th2: T helper cell type-2
VCAM-1: vascular cell adhesion molecule-1
μm: micrometer
