# Supplementary material for: Morphometric analysis of inflammation in bronchial biopsies following exposure to inhaled diesel exhaust and allergen challenge in atopic subjects
Source: Part Fibre Toxicol. 2016 Jan 13;13:2. doi: 10.1186/s12989-016-0114-z (PMC4711081; doi:10.1186/s12989-016-0114-z)
Supplement: Supplementary file 1 — Pearson correlation coefficients matrix for inflammatory biomarkers’ expression in the lung submucosa after single or co-exposure to diesel exhaust and allergen. (PDF 213 kb) [file 12989_2016_114_MOESM1_ESM.pdf]

Additional file 1

Table S1. Pearson correlation coefficients matrix for inflammatory biomarkers' expression in the lung submucosa measured after FAS (filtered air + saline) condition (upper part), and DES (diesel exhaust + saline) condition (lower part).

Variable	AA1	ECP	CD4	IL-4	CD138	NE
**AA1**	**1**	0.15 (p=0.67)	0.21 (p=0.55)	0.23 (p=0.51)	-0.07 (p=0.84)	0.05 (p=0.88)
**ECP**	0.02 (p=0.94)	**1**	0.02 (p=0.95)	-0.26 (p=0.45)	0.08 (p=0.80)	-0.18 (p=0.60)
**CD4**	0.20 (p=0.56)	0.37 (p=0.29)	**1**	-0.17 (p=0.63)	0.23 (p=0.51)	0.38 (p=0.27)
**IL-4**	0.46 (p=0.17)	-0.31 (p=0.37)	-0.15 (p=0.67)	**1**	0.53 (p=0.88)	0.37 (p=0.28)
**CD138**	-0.09 (p=0.79)	0.04 (p=0.90)	0.56 (p=0.09)	-0.25 (p=0.48)	**1**	0.13 (p=0.18)
**NE**	-0.01 (p=0.97)	0.20 (p=0.56)	0.32 (p=0.36)	-0.01 (p=0.96)	0.76 **(p<0.01)***	**1**

AA1=tryptase; ECP=eosinophil cationic protein; NE=neutrophil elastase; *p<0.05.

Table S2. Pearson correlation coefficients matrix for inflammatory biomarkers' expression in the lung submucosa measured after FAA (filtered air + allergen) condition (upper part) and DEA (diesel exhaust + allergen) condition (lower part).

Variable	AA1	ECP	CD4	IL-4	CD138	NE
**AA1**	**1**	0.07 (p=0.84)	-0.19 (p=0.58)	-0.30 (p=0.40)	0.64 **(p<0.04)***	-0.005 (p=0.99)
**ECP**	0.07 (p=0.84)	**1**	-0.26 (p=0.45)	0.61 **(p<0.05)***	-0.38 (p=0.27)	0.32 (p=0.35)
**CD4**	-0.44 (p=0.20)	-0.42 (p=0.22)	**1**	-0.31 (p=0.36)	-0.06 (p=0.86)	-0.09 (p=0.80)
**IL-4**	0.33 (p=0.34)	-0.23 (p=0.51)	0.23 (p=0.51)	**1**	-0.34 (p=0.33)	-0.23 (p=0.51)
**CD138**	-0.25 (p=0.48)	-0.37 (p=0.28)	0.54 (p=0.10)	0.19 (p=0.58)	**1**	-0.33 (p=0.34)
**NE**	0.14 (p=0.68)	-0.21 (p=0.55)	0.38 (p=0.27)	0.16 (p=0.65)	-0.13 (p=0.72)	**1**

AA1=tryptase; ECP=eosinophil cationic protein; NE=neutrophil elastase; *p<0.05.
