# Supplementary figures and images for: Morphometric analysis of inflammation in bronchial biopsies following exposure to inhaled diesel exhaust and allergen challenge in atopic subjects
Source: Part Fibre Toxicol. 2016 Jan 13;13:2. doi: 10.1186/s12989-016-0114-z (PMC4711081; doi:10.1186/s12989-016-0114-z)

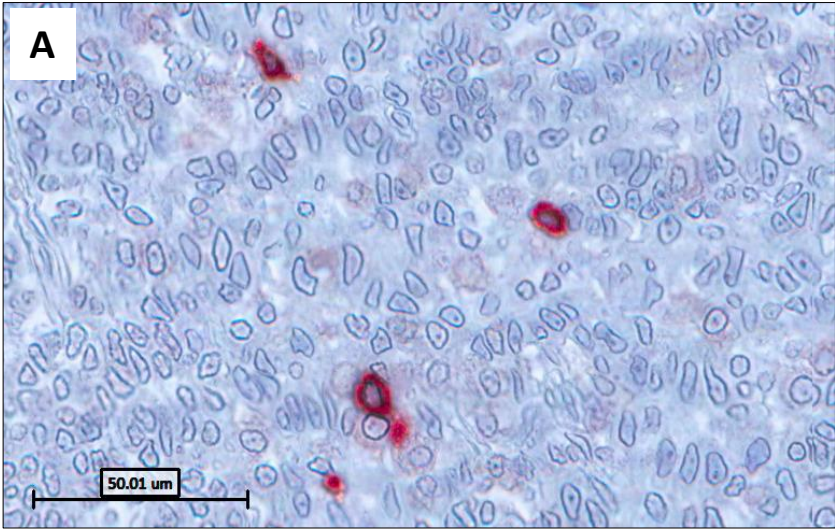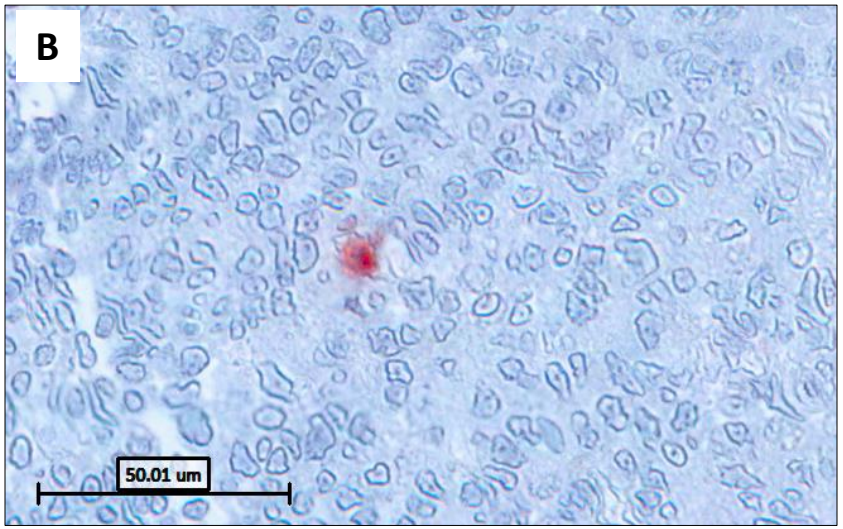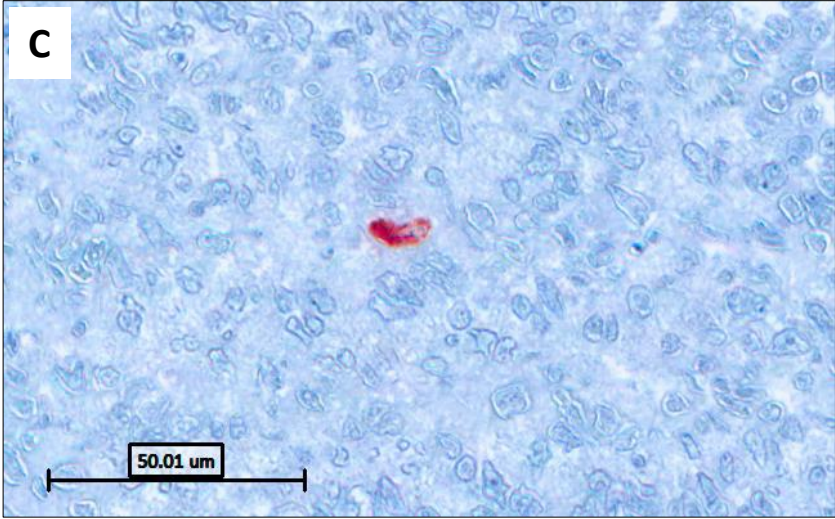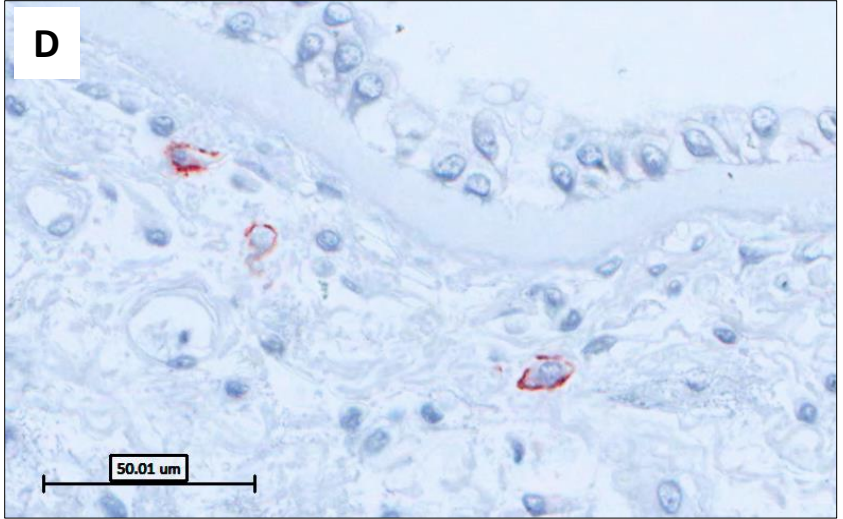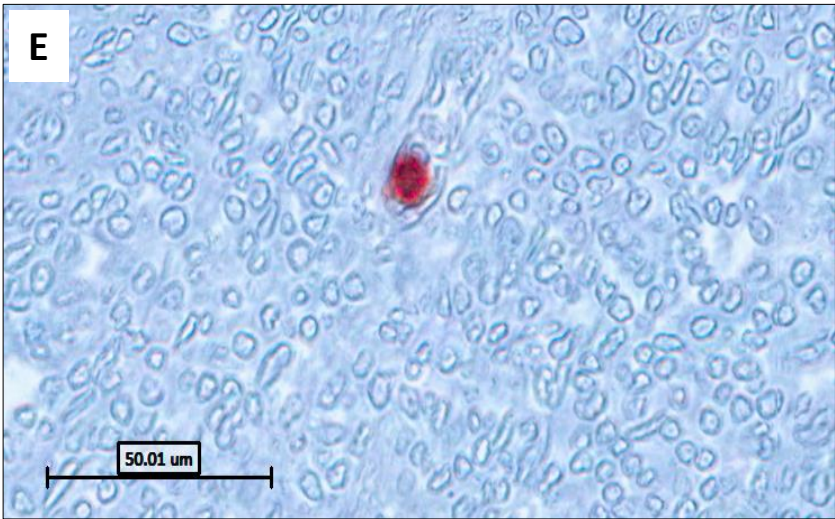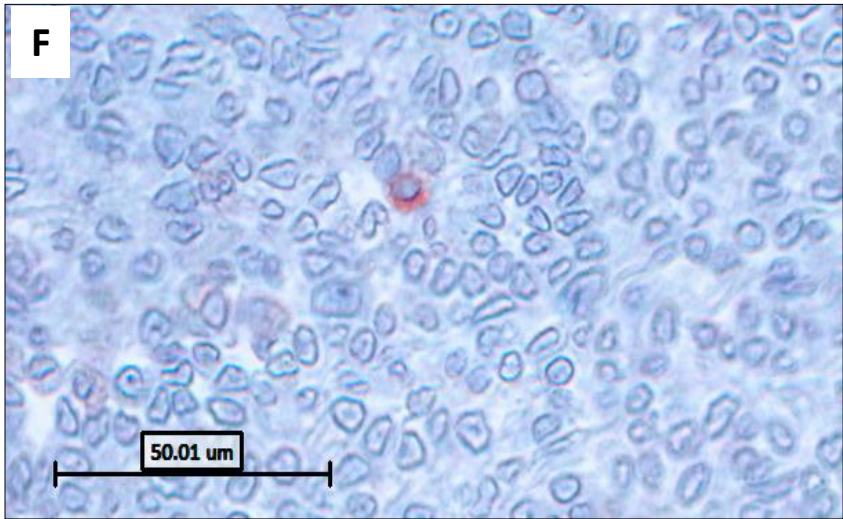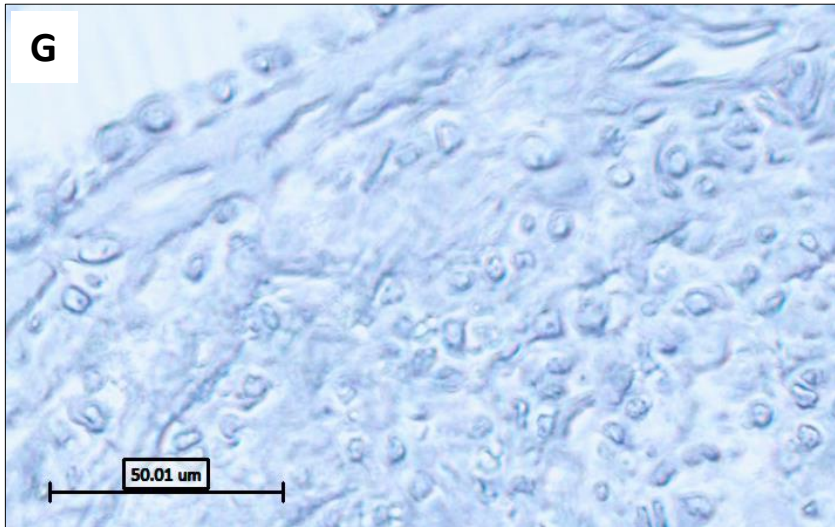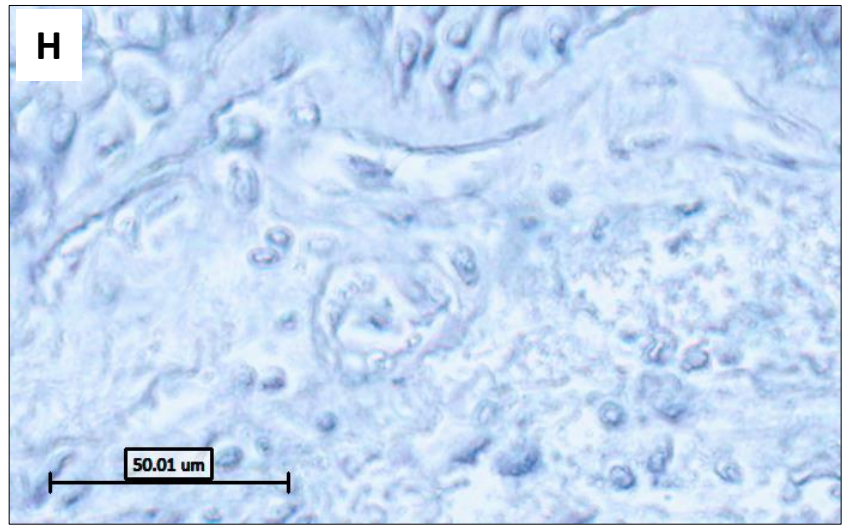

Supplement: Supplementary file 2 — Immunohistochemical staining of positive and negative controls. A) Representative 40X image of positive staining using mAb AA1 for tryptase in human tonsil tissue; B) Representative 40X image of positive staining using mAb EG2 for ECP in human tonsil tissue; C) Representative 40X image of positive staining using mAb NP57 for neutrophil elastase in human tonsil tissue; D) Representative 40X image of positive staining using mAb B-A38 for CD138 in human lung tissue; E) Representative 40X image of positive staining using mAb 4B12 for CD4 in human tonsil tissue; F) Representative 40X image of positive staining using mAb 4D9 for IL-4 in human tonsil tissue; G) Representative 40X image of isotype control using mAb mouse IgG1 (0.07 μg.mL−1) in human lung tissue; H) Representative 40X image of isotype control staining using mAb mouse IgG1 (20.0 μg.mL−1) in human lung tissue. (PDF 482 kb) [file 12989_2016_114_MOESM2_ESM.pdf]
